# Supplementary material for: Upregulation of p75NTR by Histone Deacetylase Inhibitors Sensitizes Human Neuroblastoma Cells to Targeted Immunotoxin-Induced Apoptosis
Source: Int J Mol Sci. 2022 Mar 31;23(7):3849. doi: 10.3390/ijms23073849 (PMC8998832; doi:10.3390/ijms23073849)
Supplement: Supplementary file 1 [file ijms-23-03849-s001.zip › ijms-1646017-supplementary.pdf]

International Journal of *Molecular Sciences*

MDPI

Article

Upregulation of p75NTR by Histone Deacetylase Inhibitors Sensitizes Human Neuroblastoma Cells to Targeted Immunotoxin-Induced Apoptosis

Simona Dedoni [1], Alessandra Olianas [2], Barbara Manconi [2], Maria Collu [1], Barbara Tuveri [1], Maria Elena Vincis [1], Maria C. Olianas [1] and Pierluigi Onali [1,*]

Supplementary Figures

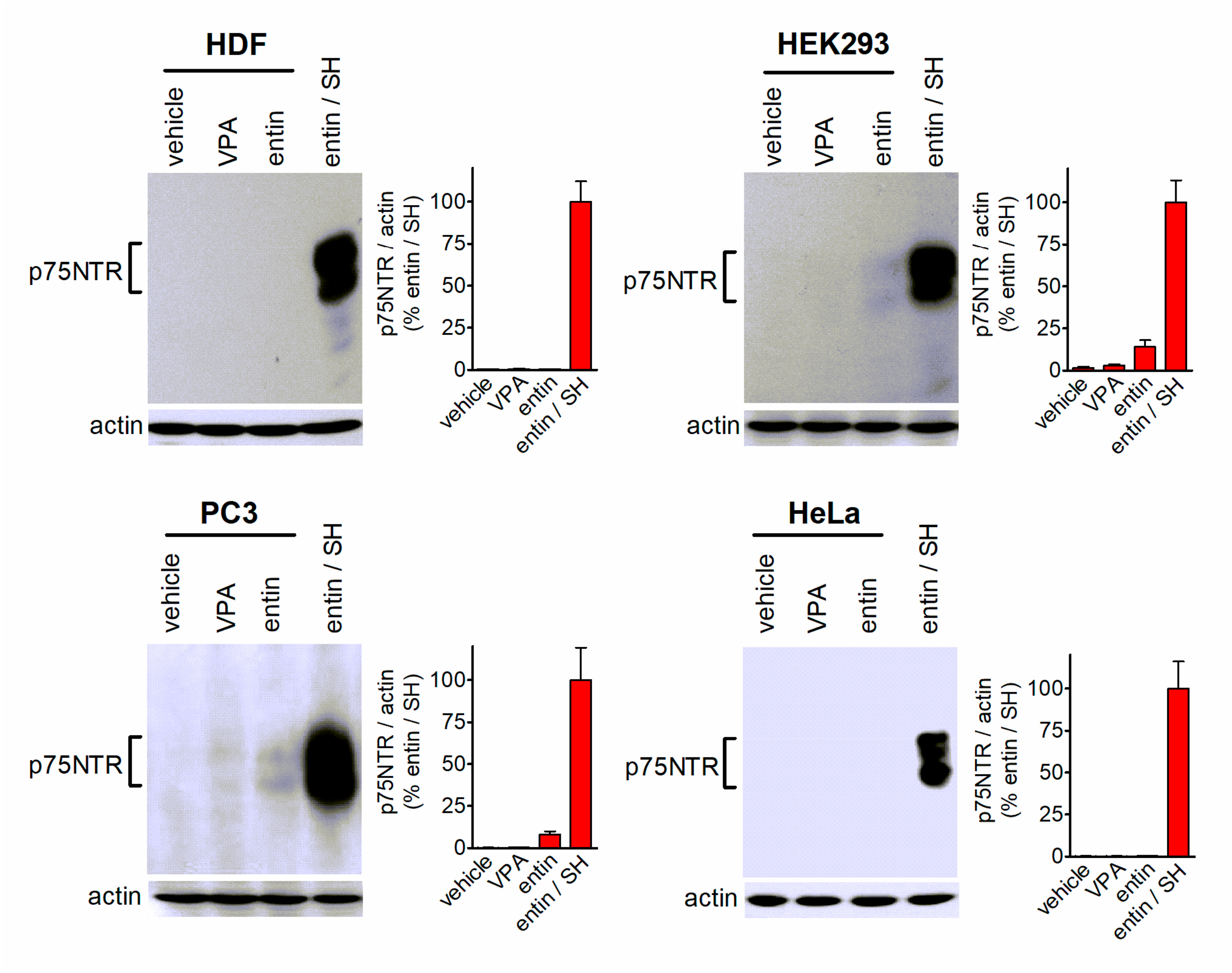

Supplementary Figure S1. Expression of p75NTR in non neuroblastoma cell lines incubated with VPA and entinostat. Human dermal fibroblasts (HDF), human embryonic kidney 293 (HEK293) cells, human prostate carcinoma PC-3 cells, and HeLa cells from human cervical carcinoma were exposed for 24 h to either vehicle, 1 mM valproic acid (VPA), or 1 μM entinostat (entin). Cell lysates were analyzed for p75NTR levels by Western blot. As a control, in each blot a sample containing an equal amount of protein obtained from entinostat - treated SH-SY5Y cells (entin / SH) was included. Values are expressed as percent of the control (entin / SH) and are the mean ± SD of three independent experiments.

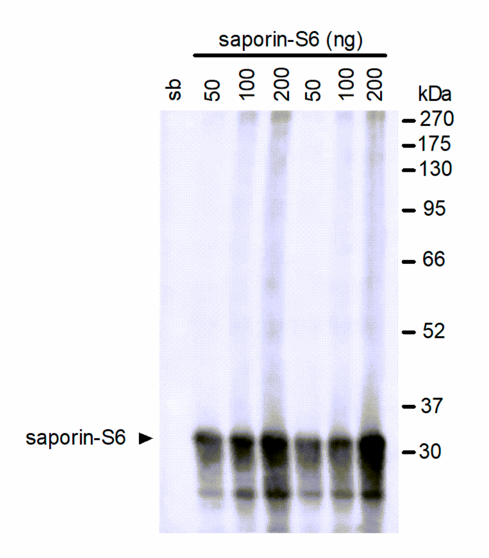

Supplementary Figure S2. Detection of saporin-S6 immunoreactivity by Western blot. Lanes were loaded with the indicated amounts of saporin-S6 and the protein immunoreactivity was analyzed by using the anti-saporin antibody PA1-18425 (Invitrogen) diluted 1:500. sb = sample buffer. The position of molecular mass standards is indicated on the right side of the blot.
